# Improved Crystallinity of Annealed 0002 AlN Films on Sapphire Substrate

**DOI:** 10.3390/ma16062319

**Published:** 2023-03-14

**Authors:** Bruno Comis Bersch, Tomàs Caminal Ros, Vegard Tollefsen, Erik Andrew Johannessen, Agne Johannessen

**Affiliations:** 1 Campus Lajeado, University of Taquari Valley, Lajeado 95914014, Brazil; 2 Campus Vestfold, University of South-Eastern Norway, Borre 3184, Norway; 3 Barcelona East School of Engineering, Polytechnic University of Catalonia, 08019 Barcelona, Spain

**Keywords:** AlN, annealing, crystallinity

## Abstract

AlN is a piezoelectric material used in telecommunication applications due to its high surface acoustic wave (SAW) velocity, stability, and mechanical strength. Its performance is linked to film quality, and one method to achieve high-quality films goes through the process of annealing. Consequently, c-orientated AlN film with a thickness of 1.1 μm deposited on sapphire was annealed at temperatures of 1100 °C and 1150 °C in a N_2_ controlled atmosphere. This was compared to annealing at 1100 °C, 1450 °C, and 1700 °C with N_2_ flow in an open atmosphere environment. Sample rotation studies revealed a significant impact on the ⍵-2θ X-ray rocking curve. A slight variation in the film crystallinity across the wafer was observed. After the annealing, it was found that the lattice parameter c was increased by up to 2%, whereas the screw dislocation density dropped from 3.31 × 10^10^ to 0.478 × 10^10^ cm^−2^, and the full width at half maximum (FWHM) of reflection (0002) was reduced from 1.16° to 0.41° at 1450 °C. It was shown that annealing in a N_2_-controlled atmosphere plays a major role in reducing the oxidation of the AlN film, which is important for acoustic wave devices where the electrodes are placed directly on the piezoelectric substrate. The face-to-face arrangement of the samples could further reduce this oxidation effect.

## 1. Introduction

Aluminum nitride (AlN) is a piezoelectric material that possesses a wide bandgap (6.2 eV), high thermal conductivity [1], one of the highest surface acoustic wave (SAW) velocities of 5760 m/s [2], low electromechanical conversion energy loss, high mechanical strength, ultraviolet transmittance, and high-temperature stability [3,4,5]. These attributes make AlN a great material for use in deep-ultraviolet light-emitting diodes (UV-LEDs), laser diodes (LDs), ultraviolet detectors, high-performance SAW devices [4,6], and signal filters [7,8]. The challenge in using AlN films for SAW device applications is the requirement of good crystallinity [9,10] which results in a better electromechanical coupling. Film deposition is performed with different tools of which reactive magnetron sputtering represents the most cost-effective option. This comes at the expense of poor crystal quality [11,12], but the crystallinity can be improved by post-deposition annealing [13]. This reduces the internal stresses of the crystal films by annihilating thread dislocations, forming dislocation loops in the crystal lattice, and merging AlN columnar structures [13,14,15,16].

The AlN films are normally grown on sapphire (common for the growth of III-V nitrides) due to its great temperature stability, mechanical strength, and low cost [17]. Sapphire has been found to be one of the best choices to minimize the formation of thread dislocations, which plays a major role in producing AlN films with good crystallinity, since there is a similarity in the respective crystal lattices and other material properties, such as the thermal expansion coefficient [6,14,18].

It was observed, from the literature review, that there was a higher focus on samples with an FWHM of the (0002) AlN rocking curve (RC) of 0.2° or lower prior to annealing, and only a few papers investigated films with a higher FWHM than 0.2° [11,19,20]. In piezoelectric films, the XRD rocking curves indicate the range of crystal orientation and dislocation density; hence it indicates the crystallinity of the film [21]. With sapphire as the substrate material, AlN is also a promising candidate for use in high-frequency telecommunication applications at elevated temperatures. The literature reports film stability up to 1200 °C in an N_2_/H_2_ atmosphere [22], although oxidation occurs already at 800 °C if kept in air [23].

Hence, this study investigates the impact of annealing AlN films at temperatures ranging from 1100 to 1700 °C in both a tube furnace offering a closed chamber with a N_2_-protected atmosphere and in an oven furnace equipped with a nitrogen stream that purges the chamber but without offering any atmospheric control (open atmosphere). The effect of the annealing on the change in crystallinity and the composition (oxidation) is recorded. The influence of the sample arranged face to face (protected by the other sample) or left with the surface facing up (unprotected) during the annealing process has also been investigated. The aim is to find ways to improve the crystallinity of AlN thin films deposited on sapphire with common laboratory equipment such as reactive magnetron sputtering.

## 2. Materials and Methods

**Figure 1:**
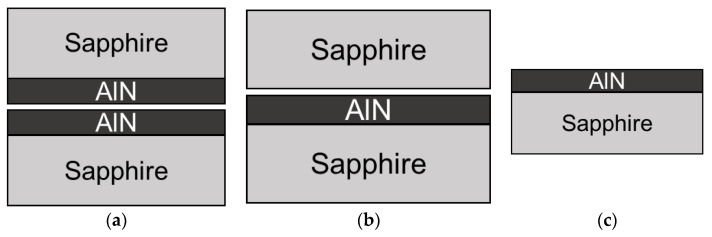
Annealing sample arrangements (**a**) face-to-face (FtF), (**b**) face-to-sapphire (FtS), and (**c**) face-opened (FO).

An AlN thin film with a thickness of around 1.1 μm was deposited onto a 4-inch (0001) single-crystal sapphire substrate using a reactive magnetron sputtering with 500 W sputtering power at a temperature of 400 °C. The N_2_/Ar gas ratio was 60/40 and the deposition pressure was 1.6 mTorr. The wafer was cut into 15 mm × 15 mm squares to facilitate the subsequent characterization and annealing process. The annealing of the samples was performed in a tube furnace under a protected atmosphere (maintained by a constant N_2_ flow of 2 L/min), at 1100 °C and 1150 °C. The annealing under a N_2_ flow in an open atmosphere was performed in a high-temperature furnace with a N_2_ flow of 15 L/min, at 1100 °C, 1450 °C, and 1700 °C. The temperature was ramped up and down at a rate of 5.5 °C/min, and the annealing temperatures were kept for 1 h, 3 h, 15 h, and 30 h, respectively, for different samples. Three different arrangements for the samples were used: face-to-face with AlN facing AlN (FtF), face-to-face with AlN facing sapphire (FtS), and an unprotected sample (FO) as shown in Figure 1, adapted from [24]. These setups were implemented to investigate the oxidation process of AlN film during annealing.

The AlN samples were characterized before and after annealing by three different techniques: (i) ⍵-2θ X-ray diffractometry (ARL Equinox 1000-XRD, Thermo Fisher, Waltham, MA, USA), equipped with a Cu-K𝛼 X-ray source, activation voltage of 40 kV, and current of 30 mA; (ii) the rocking curve of the (0002) AlN peek was characterized at 2θ = 36.2° and the omega angles used were between 15.5° and 19.5°, with a step size of 0.025° and acquisition time of 60 s; and (iii) scanning electron microscopy and energy dispersive X-ray (Hitachi SU3500-SEM and EDX, Tokyo, Japan) at a pressure of 30 Pa and acceleration potentials of 3, 5, and 10 kV. The choice of acceleration potentials was used to estimate the composition of the AlN film at different depths. The mapping of the samples, before and after annealing, was conducted with 25 measurement points in a 5 × 5 matrix pattern at 30 Pa and 5 kV. Ellipsometry (Alpha-SE, Wollam Co., Lincoln, NE, USA) was used to estimate the thickness of the AlN films. The cross-section imaging of the samples was performed using an SU8230 Hitachi SEM, Tokyo, Japan.

## 3. Results

### 3.1. Characterisation of the As-Deposited Wafer

**Figure 2:**
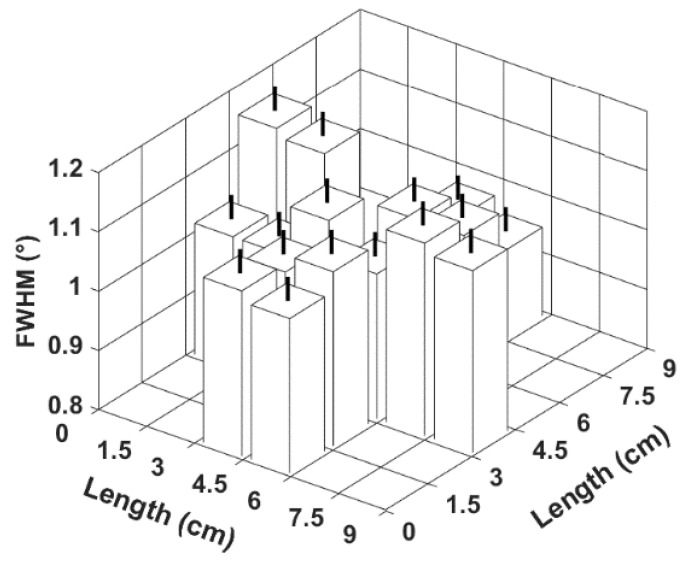
Three-dimensional histogram of the FWHM measured at different locations of the as-deposited wafer.

**Figure 3:**
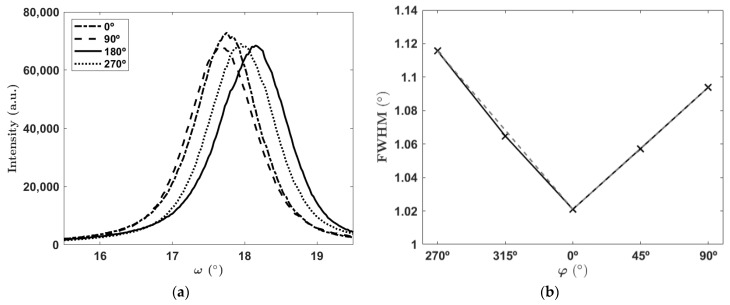
(**a**) Dependency of the rocking curves on the angle of rotation of the measured sample, (**b**) FWHMs obtained from the rocking curves in relation to the angle of rotation of the sample represented with a linear fit (dashed line) of π/2 and the measured data at π/4 angles (solid line).

After an extensive analysis of the whole wafer, a significant variation in the FWHMs can be observed (Figure 2). A region of lower FWHM is seen at the two centerlines of the wafer with variations close to 0.1°; this phenomenon could be caused by the sputtering process since AlN is not homogeneously deposited. The standard deviation seen in Figure 3a represents the error margin of each sample in Figure 2. The values of samples that have not been measured are left blank.

The mapped rocking curve, Figure 3a from the as-deposited sample, illustrates the dependency of the (0002) peak on the rotation of the sample relative to the X-ray source. This effect could be caused by some small tilt at the vertical AlN axis, slightly deflecting the X-rays and thus shifting the RC depending on the direction and angle of the tilt relative to the X-ray source. The tilt can be partially caused by the deposition setup, where during the growth process, the target is placed slightly off-center relative to the sapphire substrate. Therefore, AlN grows with slightly tilted columnar grains with respect to the sapphire’s normal axis. The FWHMs extracted from Figure 3a were 1.021°, 1.094°, 1.090°, and 1.116° for the angles 0°, 90°, 180°, and 270°, respectively, giving a standard deviation of 0.04°. The values of FWHM for 45° and 315° were comparable to the averages between both closest 𝝅/2 angles as shown in Figure 3b. Considering this variation, all the samples were marked to have the same reference position when they were analyzed before and after annealing.

### 3.2. Annealling of AlN/Sapphire Substrates

**Figure 4:**
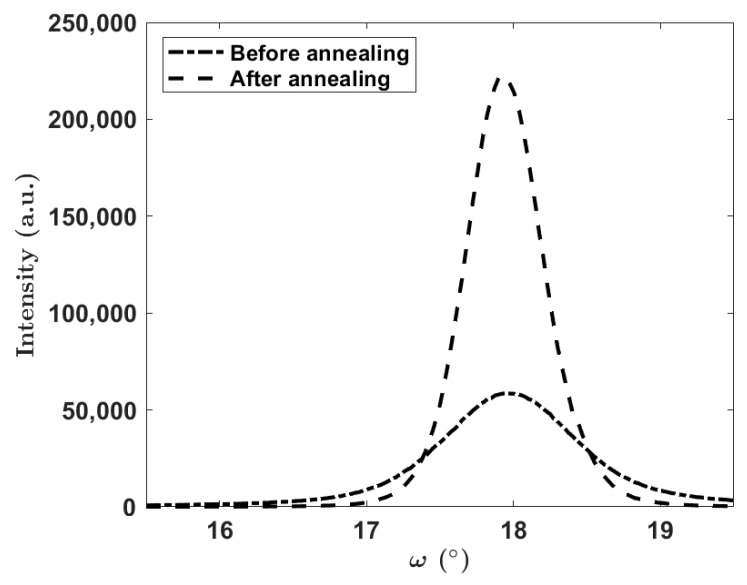
The rocking curve before and after annealing for a sample using FtF layout at 1150 °C.

Comparing the rocking curves obtained with XRD (Figure 4), a strong correlation was found between the annealing temperature and the observed improvement in crystallinity. The FWHM of the (0002) plane changed from 1.158° to 0.410°, and the intensity increased by nearly 500% at 1450 °C in the high-temperature furnace. Similar results were found at 1150 °C (Figure 4) in the tube furnace. These reductions in the FWHM suggest both a decrease in the tilt of the AlN columns and an increase in the overall grain size [25,26]. As listed in Table 1, it is inferred that a controlled atmosphere can heavily impact the crystallinity improvement as well. FO samples annealed in the high-temperature furnace became completely oxidized above 1450 °C; thus, the (0002) AlN reflection vanished, and at 1100 °C, the intensity was reduced by half. A further notable correlation was found between the sample arrangement and the FWHM enhancement: FO samples that preserved the (0002) AlN peak had a greater improvement in comparison to the FtF and FtS samples.

From Table 1, it can be observed that the intensity increased for all annealing experiments except for 1100 °C where the intensity decreased. This may be attributed to the physical processes happening at the interface between the AlN film and the Sapphire substrate, as it was observed by Solonenko et al. (2020) for AlN on a Si substrate in the transition temperature region 800–1000 °C, and was pinned to effects such as oxidation and dewetting [27].

EDX analysis of the different samples before and after annealing indicates a correlation between the temperature and the oxidation process. Diffusion is enhanced by the annealing temperature, leading to a higher degree of oxidation as the temperature is increased. Thus, reducing the partial pressure of oxygen is a prerequisite, and this can be obtained by increasing the partial pressure (concentration) of nitrogen relative to oxygen. Other gases that serve as oxygen traps, such as CO or H_2_, can be added to further reduce the partial pressure of oxygen [14,22]. Additionally, the arrangement of the samples during annealing will have an effect. Although the FO samples appeared to be highly oxidized, the FtF samples offered greater protection. Still, the experiments performed in the high-temperature furnace had an overall higher degree of oxidation than those annealed in the tube furnace. This may have been caused by the lack of adequate control of the atmospheric composition in the high-temperature furnace.

**Figure 5:**
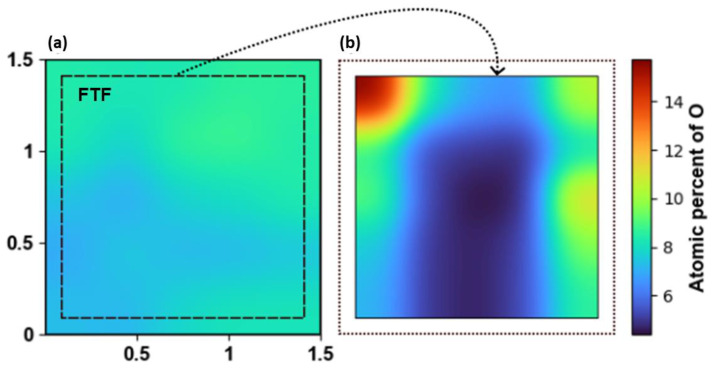
Heat map of the atomic concentration of oxygen, (**a**) before and (**b**) after annealing at 1150 °C for 15 h in tube furnace with the sample positioned FtF. The axis units in (**a**) refer to the position on the sample in cm.

The mapping of the atomic concentration of oxygen in the as-deposited sample and those annealed in the FtF arrangement at 1150 °C for 15 h are compared in Figure 5. The oxidation suppression is clearly stronger for the FtF-arranged samples, where the center surface of the film has been protected, leaving only the edges exposed to oxidation. Hence, the oxygen diffusion towards the center of the sample in the FtF arrangement depends on the pressure and composition of the protective atmosphere, the annealing temperature and time, as well as the original crystal quality as deposited. Variations could be due to the mechanisms of diffusion relative to the density of grain boundaries and imperfections in the AlN material [14,19,23,28].

**Figure 6:**
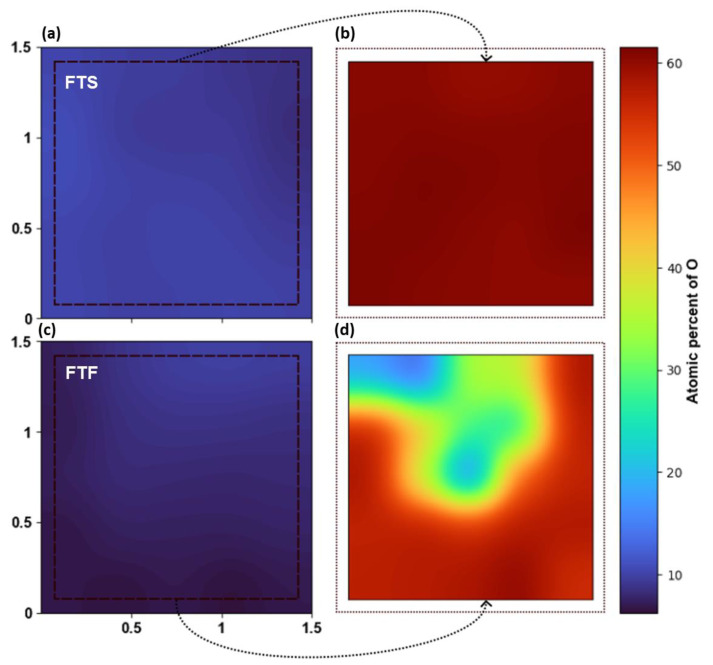
Heat map of the atomic concentration of oxygen, (**a**) before and (**b**) after annealing for FtS arranged sample; and (**c**) before and (**d**) after annealing for FtF arranged sample. Both were annealed at 1700 °C for 1 h in a high-temperature furnace. The axis units in (**a**,**c**) refer to the position on the sample in cm.

Comparing samples arranged as FtF and FtS during annealing at 1700 °C (Figure 6), it is clear that FtF arrangement had a more enhanced effect on the oxidation suppression than FtS arrangement at this high temperature.

**Figure 7:**
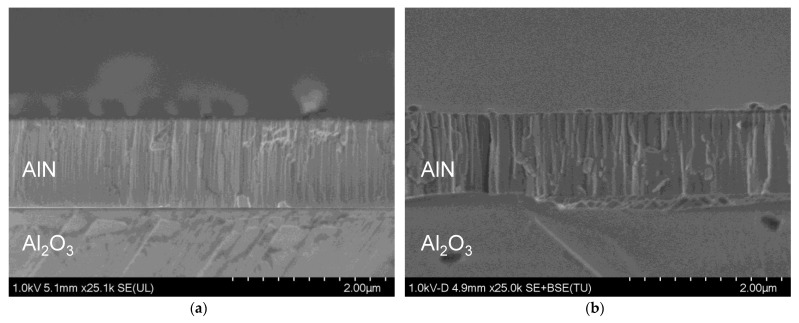
Cross-sectional SEM images of AlN films on sapphire shown (**a**) as-deposited, and (**b**) after annealing at 1150 °C in a tube furnace for 15 h.

The cross-section analysis of the as-deposited AlN film shows the presence of columnar grains in the sputtered AlN film (Figure 7a). These grains increase in size (width) after annealing as shown in Figure 7b. Such phenomena is also observed in the literature where annealed AlN films exhibited improved FWHM as discussed in the beginning of Section 3.2.

## 4. Discussion

From the obtained experimental data, the estimated lattice parameter *c* for the as-deposited AlN samples was 4.9443 Å. Table 2 shows the values of the lattice parameter *c* for each annealing and sample arrangement. Hence, from Table 2, there is a tendency that the *c* lattice constant of the AlN film increased as a result of annealing. This was probably caused by a relaxation of the compressive stresses that existed in the as-deposited sample [12] and where the addition of thermal energy shifted the atomic bonds to a lower energy state, thus increasing the c lattice parameter and the film thickness [5].

As-deposited AlN films are known to possess various types of defects such as threading dislocations, dislocation loops, basal stacking faults, and inversion domains [29,30,31]. Some of these defects can be visualized using SEM and TEM imaging, but others such as dislocation densities can also be estimated from the rocking curve. Screw edge and mixed dislocation can be estimated from (0002) and $\left(10\bar{1}2\right)$ RC (1) (2) [32]:

(1) $$\rho_{s}=\frac{\beta_{\left(0002\right)}^{2}}{2\pi \ln\left(2\right){\left|b_{c}\right|}^{2}}$$

(2) $$\rho_{e}=\frac{\beta_{\left(10\bar{1}2\right)}^{2}}{2\pi \ln\left(2\right){\left|b_{a}\right|}^{2}}$$

where |**𝑏**_𝑐_| and |**𝑏**_𝑎_| are the burgers vectors of each dislocation, and (0002) and 𝛽 ($10\bar{1}2$) are the FWHM values, in radians, for the (0002) and $\left(10\bar{1}2\right)$ RC, respectively. Equation (2) relates the FWHM of the (0002) RC with the screw-type dislocation since the screw dislocations density (𝜌_𝑠_) affects the out-of-plane rotation; this is seen in the symmetric reflections from (0002), (0004), and (0006). Equation (2), on the other hand, relates the FWHM of skew-symmetric reflections, $\left(10\bar{1}2\right)$ RC for example, with edge dislocation density (𝜌_𝑒_) as a result of the tilt and twist of the change in the lattice laying in-plane rotation [32,33]. The screw dislocation density found for the as-deposited samples was 3.22·10^10^ ± 3.60 × 10^9^ cm^−2^, the values for the 𝜌_𝑠_ after the annealing of the samples arranged in the FtF position are stated in Table 2.

Previous work related to the annealing of AlN films is summarized in Table 3. The selection is based on the deposition method used, film thickness, annealing parameters, and the corresponding FWHM. This gives us a comparative analysis of the different methods used and how they impact the crystallinity, as well as visualizing the as-deposited quality of the AlN film used.

## 5. Conclusions

Annealing at temperatures above 1100 °C has been found to improve the crystallinity of AlN thin films that have been deposited on sapphire substrates using RF magnetron sputtering. An evident correlation between the increase in the annealing temperature and the improvement of the FWHM has been found. The danger of sample oxidation can be reduced by maintaining an adequate protective atmospheric composition and by having a good arrangement of the annealed samples. Arranging the samples as FtF and FtS has been shown to heavily suppress the oxidation of the AlN films at high temperatures compared to FO. This will permit an increase in the maximum temperature and time that the film can be subject to during the annealing process, which is important to improve the crystal quality of the AlN film. The samples studied in this work are comparably small (15 mm × 15 mm), and even the optimal FTF arrangement showed signs of oxidation penetrating from the edges during annealing. Besides considering the FtF arrangement, the oxidation could be further reduced by using ovens with a protective inert atmosphere. This would be of high interest when the annealing process is implemented for larger sample sizes (such as 3- or 4-inch wafers). Even if oxidation occurs around the edge of these wafers, the central region will still be protected. Hence, the results presented show the significance of applying high-temperature annealing in order to gain improved crystallinity. Further studies should be carried out to optimize the annealing processes of sputtered AlN films in order to achieve comparable quality to that obtained with deposition tools designed explicitly for AlN film deposition.

## Figures and Tables

**Table 1:** Annealing impact on the rocking curve from the test samples.

Arrangement	Furnace Type	Annealing Conditions		FWHM (002), °		Change in FWHM, $\Delta f/f_{0},\;\%$	Change in Intensity $\Delta I/I_{0},\;\%$
		*T*, °C	*t*, h	As-Deposited	Annealed		
FO	TF ^a^	1100	15	1.044	0.928	−11.1%	−67.1%
FtF		1100	15	1.017	1.001	−1.5%	−8.0%
			45	1.017	0.975	−4.2%	−14.5%
FtF		1150	15	1.060	0.602	−43.2%	+280.5%
FtF	HTF ^b^	1100	1	1.144	1.034	−9.6%	−54.2%
FtF		1450	1	1.158	0.410	−64.6%	+486.5%
FtS		1700	1	1.125	0.535	−52.5%	+250.7%
FtF		1700	1	1.107	0.598	−46.0%	+208.3%
FO		1700	3	1.055	-	-	-

^a^ Tube furnace. ^b^ High-temperature furnace.

**Table 2:** Lattice parameter *c* as a function of different annealing parameters.

Sample	Arrangement	Annealing Time, (h)	Instrument	Lattice Parameter *c* (Å)	SDD (cm^−2^)
as-deposited				4.9443	3.31 × 10^10^
1100 °C	FtF	1	HTF	4.9559	3.06 × 10^10^
	FO	15	TF	4.9559	2.56 × 10^10^
	FtF	15		4.9519	2.25 × 10^10^
	FtF	45		4.9519	2.38 × 10^10^
1150 °C	FtF	15	TF	4.9559	1.03 × 10^10^
1450 °C	FtF	1	HTF	4.9599	4.78 × 10^9^
1700 °C	FtS	1	HTF	4.9483	8.13 × 10^9^
	FtF	1	HTF	4.9599	1.02 × 10^10^

**Table 3:** Literature review on the annealing of AlN films.

Deposition Method (Substrate)	AlN Thickness (nm)	Annealing Temperature (°C)	Annealing Time (h)	Sample Arrangement	Annealing Atmosphere	(0002) FWHM	Ref
LPMOCV (Al_2_O_3_)	50	As-dep. 1070	0.25	-	-	0.47°→ 0.32°	[34]
Sputtering (Al_2_O_3_)	170	As-dep. 1600 1650 1700	1	FtF	N_2_	0.148°→ 0.014° 0.017° 0.013°	[24]
	340	As-dep. 1600 1650 1700				0.653°→ 0.039° 0.042° 0.014°	
Sputtering (Al_2_O_3_)	1000	As-dep. 1500 1600 1650	1	FtF	Low-pressure N_2_	0.200°→ 0.112° 0.115° 0.121°	[11]
	200	As-dep. 1500 1600 1650				0.045°→ 0.034° 0.031° 0.031°	
Sputtering (Al_2_O_3_)	1300	1000	As-dep. 3 6 12 21	-	Air	0.5°→ 0.438° 0.407° 0.297° 0.267°	[23]
		900 800 700				Unchanged: 0.52°–0.48°	
MOVPE (Al_2_O_3_)	300	As-dep. 1500 1550 1600 1650 1700 1750	2	-	N_2_-CO	0.051°→ 0.018° 0.018° 0.019° 0.019° 0.038° 0.127°	[14]
MOVOP (Al_2_O_3_)	300	1700	As-dep. 0.5 1 2 4	-	N_2_-CO	0.019°→ 0.008° 0.008° 0.012° 0.010°	[35]
MOCVD (Al_2_O_3_)	540	As-dep. 1550 1600 1650 1700 1750	1	FtF	N_2_	0.034°→ 0.032° 0.025° 0.021° 0.016° 0.018°	[36]
Sputtering (Al_2_O_3_)	1100	As-dep. 1150	15	FtF	N_2_ (Tube furnace)	1.060°→ 0.602°	This work
		As-dep. 1450	1		N_2_ (High-temp furnace)	1.158°→ 0.410°	

^“→” indicates FWHM after annealing.^

## Data Availability

Not applicable.
